# Symptomatic Cavum Vergae Cyst in a Geriatric Patient: A Report of a Rare Case and Conservative Management Approach

**DOI:** 10.7759/cureus.57907

**Published:** 2024-04-09

**Authors:** Plamen Penchev, Petar-Preslav Petrov, Vladislav Velchev, Andrey Velev

**Affiliations:** 1 Faculty of Medicine, Medical University of Plovdiv, Plovdiv, BGR; 2 Department of Anatomy, Histology and Embryology, Medical University of Plovdiv, Plovdiv, BGR; 3 Department of Neurology, Military Medical Academy Hisarya, Hisarya, BGR

**Keywords:** headache, cavum septum pellucidum, intracranial cyst, cavum vergae cyst, case report

## Abstract

Cavum vergae (CV) cysts constitute a small proportion of intracranial cysts, and although generally asymptomatic, there are occasional cases where they might exhibit clinical manifestations. We present a clinical case of a 79-year-old female patient who had a clinical manifestation of headache on the occipital side of the head with irradiation to the shoulder girdle as well as numbness, dizziness, visual impairment, sleep disturbances, and tingling in the hands for three months. Vertigo and rightward staggering had been experienced for two weeks. On physical examination, it was discovered that there was smoothed physiological lordosis, restricted and painful movements, and paravertebral muscle rigidity in the cervical region. The patient had bilaterally reduced biceps and triceps reflexes, painful Erb’s points, and hypesthesia over the C5 and C6 dermatomes on the right side. The patient had decreased coordination and displayed staggered movement to the right. A CT scan discovered dilated subarachnoid spaces of the convexity and a CV cyst. The patient was prescribed conservative therapy consisting of etoricoxib oral at a dosage of 2 × 60 mg for seven days, tolperisone hydrochloride orally at a dosage of 2 × 150 mg for seven days, pregabalin 75 mg, one pill in the evening for seven days, ozoid (a gel containing ozone) for external application, and vinpocetine 2 × 10 mg orally for two months. Following the conservative treatment, the patient exhibited improvement in her symptoms and no longer had challenges carrying out her daily tasks. Furthermore, six months after the therapy, the patient did not experience any symptoms. Long-term follow-up will be conducted in cases of symptom recurrence or cyst enlargement.

## Introduction

The septum pellucidum (SP) is a thin, translucent, and triangular bilayer membrane that serves as a barrier between the right and left frontal horns and the lateral ventricles of the brain. The structure extends from the front part of the corpus callosum to the structure of the fornix, with a width ranging from 1.5 to 3.0 mm. The cavum SP (CSP) and cavum vergae (CV) are enduring membrane structures within the adult brain that arise due to the incomplete closure of the membranous leaves of the septum pylori. CV refers to the posterior extension of the SP [1].

The CSP cyst, the CV cyst, and the cavum velum interpositum cyst are some of the different types of benign midline anterior cerebral cysts. The cysts are considered pathological when they exhibit symptoms, which vary depending on their size. CV cysts are rare lesions with an incidence of 2.32% in adults. The majority of these cysts do not exhibit symptoms, although there are rare cases where they may manifest clinical symptoms. The most prevalent symptom is headache, although these lesions can present with a wide range of symptoms such as neurological deficit, ataxia, seizures, syncope, and visual and sensory irregularities [1-3].

The main purpose of this case report is to emphasize the significance of physicians considering a CV cyst as one of the various types of cystic lesions that may affect the SP. Moreover, we recommend selecting an initial conservative management approach as the preferable treatment plan if there is no blockage of CSF, compression of tissues, or changes in mental status. We could consider surgical intervention as a treatment option if conservative approaches prove ineffective.

## Case presentation

**Figure 1:**
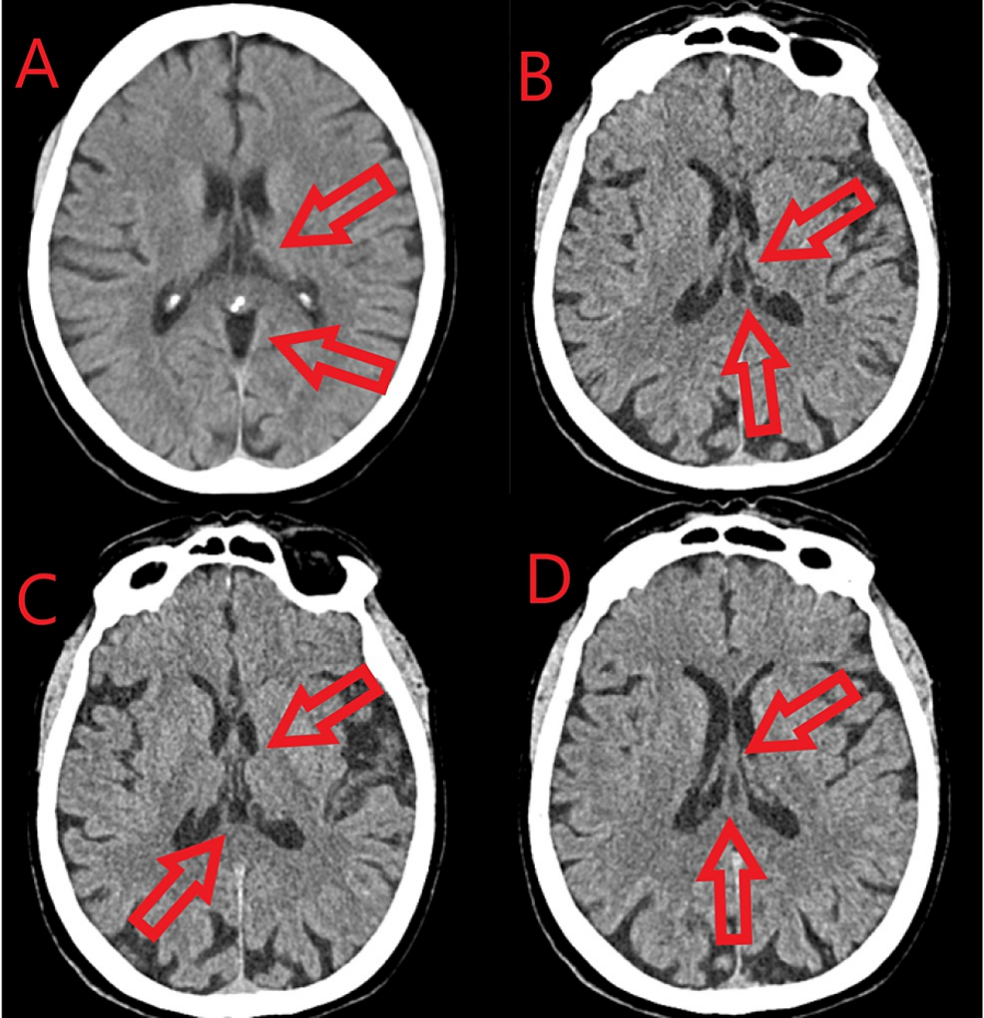
CT scan (axial plane) (A, B, C, D) Dilated subarachnoid spaces of the convexity and a CV cyst CV, cavum vergae

We present a clinical case of a 79-year-old female patient who manifested headaches on the occipital side of the head, with irradiation to the shoulder girdle, as well as numbness, dizziness, visual impairment, sleep disturbances, and tingling in the hands for three months. No changes in mental status were observed. During this period, she received treatment with piracetam and betahistine, but the medications did not provide any effect. Over the past month, her movements became limited, and she tended to drop objects. Symptoms worsened in cold and damp weather with physical activity and prolonged standing, resulting in increased difficulty in performing daily activities. The patient experienced vertigo and rightward staggering for two weeks. Physical examination revealed smoothed physiological lordosis, restricted and painful movements, and paravertebral muscle rigidity in the cervical region. Bilaterally reduced biceps and triceps reflexes, painful Erb’s points, and hypesthesia over the C5 and C6 dermatomes on the right side were noted. The patient displayed decreased coordination and staggered movements to the right. A CT scan revealed dilated subarachnoid spaces of the convexity and a CV cyst (Figure 1).

The patient was prescribed conservative therapy consisting of etoricoxib oral at a dosage of 2 × 60 mg for seven days, tolperisone hydrochloride orally at a dosage of 2 × 150 mg for seven days, pregabalin 75 mg, one pill in the evening for seven days, ozoid (a gel containing ozone) for external application, and vinpocetine 2 × 10 mg orally for two months. Following the conservative treatment, the patient exhibited improvement in her symptoms and no longer had challenges carrying out her daily tasks. Furthermore, six months after the therapy, the patient did not experience any symptoms. Long-term follow-up will be conducted in cases of symptom recurrence or cyst enlargement.

## Discussion

Numerous studies fail to clearly state the duration of clinical progression for individual patients with CSP and/or CV cysts, according to Simonin and Lind. Nevertheless, the majority of cases exhibit a progression ranging from one month to three years [3]. Our patient’s clinical presentation demonstrated a three-month progression, aligning with the findings of Simonin and Lind.

In a recent meta-analysis with 368 patients conducted by Kryukov et al., the following symptoms were analyzed: headache (n = 184; 50%) and convulsive syndrome (n = 87; 23.6%); reduced intelligence/delayed psychomotor development (n = 74; 20.1%); mental disorders (n = 58; 15.8%); dizziness, nausea, and vomiting (n = 40; 10.9%); impaired consciousness (n = 36; 9.8%); gait disorders (n = 33; 9%); visual impairment (n = 31; 8.4%); optic nerve swelling (n = 17; 4.6%); cranial nerve dysfunction (n = 15; 4%); and hydrocephalus (n = 61; 16.6%) [4]. In the present study, the patient exhibited clinical manifestations including headache, dizziness, visual impairment, and sleep disturbances, therefore aligning with the findings of Kryukov et al.

Das and Dossani recommend surgical intervention when there is a blockage of CSF flow in the foramen of Monro, direct compression of adjacent tissues, or alterations in mental state [5]. Our case did not show any indications of CSF blockage, compression of nearby tissues, or alterations in mental state.

Several studies state that CSP and CV cysts are typically regarded as accidental observations; however, certain ones may have pathogenic effects. Several mechanisms can be used to justify this phenomenon. These mechanisms include the obstruction of the interventricular foramen, which can lead to hydrocephalus and/or increased intracranial pressure. Additionally, compression of the hypothalamic-septal triangle can result in neuropsychiatric symptoms, while compression of the optic chiasm and its pathways can also contribute to this condition. Furthermore, chronic deep venous involvement can cause progressive focal deficits [6-9]. Despite being congenital, we were unable to identify any etiological factor that could account for its clinical manifestation in the elderly.

## Conclusions

This case report emphasizes the importance of considering CV cysts in the differential diagnosis of intracranial cystic lesions appearing on the SP, especially in older individuals who exhibit neurological symptoms. Although these cysts tend to be accidental findings, they might result in substantial morbidity if they exhibit symptoms. In instances where there is an absence of cerebrospinal fluid blockage or compression of nearby tissues, conservative therapy, as seen in our patient, has proven to be effective in reducing symptoms without requiring surgical intervention. The long-term follow-up of symptom recurrence or cyst expansion is of paramount significance. Additional investigation is necessary to have a more comprehensive understanding of the natural progression and most effective approaches for the management of CV cysts.
